# Implementing clinical pharmacy activities in hospital setting in Vietnam: current status from a national survey

**DOI:** 10.1186/s12913-022-08242-5

**Published:** 2022-07-07

**Authors:** Phuong Thi Xuan Dong, Hieu Trung Trinh, Duy Huu Nguyen, Son Tu Nguyen, Van Thi Thuy Pham, Ha Bich Ngo, Susan Hua, Shu Chuen Li, Huong Thi Lien Nguyen

**Affiliations:** 1 Department of Clinical Pharmacy, Hanoi University of Pharmacy, 13-15 Le Thanh Tong Street, Hanoi, Vietnam; 2 Department of Pharmacy, Friendship Hospital, Hanoi, Vietnam; 3 School of Biomedical Sciences and Pharmacy, College of Health, Medicine and Wellbeing, University of Newcastle, Callaghan, NSW 2308 Australia; 4 Medical Services Administration, Ministry of Health, Hanoi, Vietnam

**Keywords:** Clinical pharmacy, Pharmacy practice, National survey, Vietnam

## Abstract

**Background:**

Clinical pharmacy activities have evolved over the past decades contributing to all stages of the patient care process, especially in the hospital setting. However, these practice roles may differ to a significant extent depending on the healthcare policy of countries. In Vietnam, the magnitude of adopting clinical pharmacy activities in hospital settings throughout the country is still unknown since these activities have been implemented. This study aimed to ascertain the current status of clinical pharmacy activities performed within the Vietnamese hospital setting.

**Methods:**

A nation-wide survey was conducted from December 2017 to January 2018. Two online questionnaires, one for the Heads of Pharmacy Department and one for clinical pharmacists, were designed based on the national legal regulations about implementing clinical pharmacy activities in the hospital setting. These questionnaires were sent to all hospitals and healthcare facilities with a department of pharmacy.

**Results:**

A total of 560 Heads of Pharmacy and 574 clinical pharmacists participated in the study, representing a response rate of 41.2%. Among the participating hospitals, *non-patient specific* activities were implemented widely across all hospital classes, with pharmacovigilance, medication information, and standard operating procedures development implemented in ≥88% of all hospitals. In contrast, there was a significant variation in the level of implementation of *patient-specific activities* among hospital classes. With *activities* such as medication counselling, monitoring of adverse drug reactions, and obtaining patient’s medication histories provided at a considerably lower level in between 49 and 57% of hospitals.

**Conclusion:**

Clinical pharmacy activities have been initiated in most of the surveyed hospitals. In general, clinical pharmacy is more established in higher-class hospitals in Vietnam. However, the current implementation status is focused on non-patient-specific activities, while patient-oriented activities remained insufficiently established.

**Supplementary Information:**

The online version contains supplementary material available at 10.1186/s12913-022-08242-5.

## Background

Clinical pharmacy is a health science discipline in which pharmacists provide pharmaceutical care that optimizes medication therapy and promotes health, wellness, and disease prevention [1]. Clinical pharmacy services have been widely proven to reduce adverse drug reactions (ADRs) and hospital readmissions, improve medication adherence and appropriateness, and enhance clinical outcomes for patients [2, 3]. With this practice mode, the responsibilities of pharmacists are no longer limited to drug manufacturing and supply. Instead, their role has significantly expanded to incorporate a number of clinical pharmacy services across various clinical settings, including in many patient care areas in hospitals [1, 2, 4]. While clinical pharmacy services are well-established in many developed countries [5, 6], these practice roles may differ to a significant extent depending on the healthcare policy and resources in other countries [7–9].

In Vietnam, a lower-middle-income country in Southeast Asia, with a high-pressure healthcare system and a low ratio of healthcare workers per capita, the extent of clinical pharmacy development in healthcare facilities is still not fully explored. For almost 30 years, the Ministry of Health (MOH) in Vietnam has issued consecutive “circulars” and “decisions” related to clinical pharmacy areas. Examples of these documents include Pharmacy and Therapeutic Committees (1997), Medicines Information Centers in Hospitals (2003) [10], and MOH’s Regulation Circular 31 (2012) – the latter was the first legal framework for implementing clinical pharmacy in Vietnamese hospitals [11]. Most recently, clinical pharmacy was defined explicitly in the updated Pharmaceutical Law (2016) [12], and included administrative rules related to clinical pharmacy. The actions of the health authorities demonstrate that clinical pharmacy is becoming more important and is gradually recognized and accepted in hospitals and by clinical leaders in Vietnam.

Along with significant policy changes, clinical pharmacy education and training in Vietnam have improved since the 2010s. Pharmacy schools began changing their curricula to include a greater emphasis on patient-centered care and clinical practice. The 2007–2012 project “Strengthening the training quality of clinical pharmacists in Vietnam,” in which six Vietnamese schools of pharmacy collaborated with Dutch, Thai, and Indonesian institutions, has integrated clinical pharmacy as a specialization into existing pharmacy programs. In 2012, the Ministry of Education and Training issued BPharm curriculum reform [13], which required pharmacy schools to provide a specialization in clinical pharmacy. This was a step in preparing well-trained human resources for implementing clinical pharmacy activities in Vietnamese hospitals.

Following these initiatives, hospitals are obliged to carry out clinical pharmacy services according to the Law. However, the Law just provides basic requirements, including functions, responsibilities, and organizational structure of clinical pharmacy services (CPS) at hospital facilities, but not the specifics on the extent to which CPS must be implemented. Furthermore, it has not yet established quality assurance criteria for clinical pharmacy services as well. As a result, clinical pharmacy services have been provided with substantial variation in scope and scale across Vietnamese hospitals, depending on their needs, workforce, and facilities. These services were broadly described in a few small-scale studies with limited information detailed. These recent studies on the clinical pharmacy services in Hanoi and Ho Chi Minh City (two of the biggest cities in Vietnam) [14–16] reported that the most described clinical pharmacy activities were non-patient specific activities, with the most common being the provision of drug information, participation in pharmacovigilance activities, and research of medication usage. Direct-patient care activities were limited and varied widely among hospitals. These studies also highlighted that the main obstacles faced by most hospitals were insufficient workforce and lack of qualified clinical pharmacists [14, 15].

Nevertheless, it should be noted that these studies were only limited to one city [14, 15]. Therefore the results cannot be extrapolated to identify clinical pharmacy services across the country after promulgating and implementing the official regulations. To evaluate the impact of the legal requirements, there is a need to perform a more comprehensive study to provide more generalizable information about the current status of the practice of clinical pharmacy in Vietnamese hospitals.

## Methods

### Aim

The aims of this study was to assess the workforce involved in providing clinical pharmacy activities in Vietnamese hospitals and to describe the current extent of clinical pharmacy activities performed within the hospital setting. The differences in clinical pharmacy activities between the hospital classes were also compared in this study. The key reason for conducting the study was to understand the necessary future changes required and support strategies needed in Vietnamese hospitals to improve the implementation of clinical pharmacy services.

### Study design and setting

This study was a part of a project supported by the Department of Medical Services Administration (DMSA) from the Ministry of Health (MOH) to investigate the current status of clinical pharmacy services and medication information services in Vietnamese hospitals. The project was conducted in the context of the development of the Decree of Clinical Pharmacy and the National Guideline of Clinical Pharmacy Services to be released to understand the extent of implementation of clinical pharmacy services throughout the whole country. The study methods have previously been published in another article about medication information services by the research group [17].

In brief, a national cross-sectional survey was conducted in Vietnam, a middle-income country in Southeast Asia with a population of 94.6 million (2017). All hospitals with a pharmacy department were invited to this study, with a total number of 1359 according to the Health Statistics Yearbook 2017 [18].

### Definition of hospital class

According to the regulations of the Ministry of Health of Vietnam, all hospitals are categorized in descending order as Special Class, Class 1, Class 2, Class 3, or Class 4 based on the following predefined criteria [19–21] – (i) location, function, mission, (ii) scale and content of operation, (iii) technical expertise, infrastructure, and (iv) medical equipment. The classification of hospitals is the basis for technical classification and development orientation of hospital activities over time, including clinical pharmacy activities. Therefore, the extent of clinical pharmacy implementation was analyzed based on hospital classes in this study.

### Design of the questionnaires

According to the clinical pharmacy regulations of the Ministry of Health [10, 11, 22], the activities of clinical pharmacists in the hospital setting are organized into two main categories:

- *Non-patient specific activities* including participation in hospital committees, development of guidelines and protocols for medication use, development of treatment guidelines in collaboration with medical and nursing teams in the departments involved, participation in pharmacovigilance activities, participation in pharmacy research, and provision of medication information to healthcare professional staff.
- *Patient-specific activities (i.e., pharmaceutical care activities or patient-centered care activities)* comprised of the patient-related stream (e.g., obtaining medication history and medication counseling for patients) and the treatment-related stream (e.g., ward rounds and medication reviews, and working with physicians in the optimization of therapy).

Therefore, two separate questionnaires were developed to explore the current extent of each group of activities implemented in Vietnamese hospitals. The first questionnaire *(Part 1 Survey – Additional file* *1**),* which was to be completed by the Head of the Pharmacy Department of each hospital, consisted of multiple-choice questions to solicit workforce information and extent of non-patient specific activities. The second questionnaire *(Part 2 Survey – Additional file* *2**)*, which aimed to obtain the extent of *patient-specific activities* provided by clinical pharmacists, was answered by all clinical pharmacists willing to participate. The survey questionnaires were designed corresponding to the clinical pharmacy activities required by Circular No. 31 and clinical pharmacy literature [14, 23]. Although there was no formal validation, the questionnaires were reviewed and pilot-tested for eliminating errors and user-friendliness by five clinical pharmacists in Hanoi hospitals. Four members of the research team and two clinical pharmacists from a public hospital in Hanoi checked face and content validity of the draft questionnaires before they were finalized and the online platforms were created.

### Definitions of patients-specific activities

While *non-patient specific activities* are clearly defined and accompanied by practice guidelines (for example, drug information, pharmacovigilance, Drug and Therapeutics Committee), *patient-specific activities* of clinical pharmacists have not been defined explicitly in regulations in Vietnam, specifically new terms such as “medication review,” “ward round,” and “co-participation with physicians in therapy optimization”. In the current study, these terms were defined as follow:

“Ward round” refers to the clinical pharmacist’s presence in the clinical department, with or without the doctor present, to examine the patient’s medication use, progress, and clinical/subclinical response of the patient.

“Medication review” denotes the activities of the clinical pharmacist in evaluating the appropriateness of the patient’s prescribed medication using information retrieved from their medical record/prescription record. Circular 31 regulates this activity in combination with the process of ward round. The term “co-participation with physicians in therapy optimization” in Circular 31 refers to the activity that occurs following clinical pharmacists’ identification of drug-related problems in prescribing, the clinical pharmacists actually provide recommendations to physicians regarding medication prescribing in order to optimize patient’s therapy.

### Data collection

Data collection for the questionnaires was supported and facilitated by the Vietnamese Department of Medical Services Administration (DMSA) from the Ministry of Health (MoH). First, an invitation letter was delivered using the Department’s internal electronic portal, which automates the distribution of the letters to hospitals under the Department’s administration– including all 63 Provincial Health Bureaus. Furthermore, the Provincial Health Bureaus were asked to send the invitation letter to the board of directors of all hospitals under their direct administration. Usually, such documents are received by the hospital’s department of general administration and then transferred to the hospital director, who will assign them to the appropriate departments, in this case, the Department of Pharmacy. The hospitals that accepted to participate in the study then used the link of the Online Form attached to the invitation letter to answer the survey. Online forms (created using Google Form®) were available from December 2017 to January 2018. The first questionnaire was responded by the Heads of the Pharmacy Department, with each hospital providing only one response. The second questionnaire was responded by all clinical pharmacists willing to participate. The questionnaires of the survey were developed and distributed in Vietnamese.

### Data analysis

After receiving the results, the data were then analyzed using Stata 13.0. All data were described as percentage (categorical data) or mean with standard deviation (data with normal distribution) or median with interquartile range (data with non-normal distribution), where appropriate. The workforce characteristics and current status of clinical pharmacy activities were compared among hospitals by class. The Likert scale [24] was employed to assess the extent of provision of clinical pharmacy activities, with 1 = never/don’t have; 2 = rarely; 3 = sometimes; 4 = usually; 5 = always. To compare the level of implementation between hospital classes, the Kruskal–Wallis test (for non-normally distributed quantitative variables), the Chi-square test, and Fisher’s Exact test (for categorical variables) were applied, followed by post hoc pairwise comparisons.

### Ethics approval

This study was approved and supported by the Department of Medical Services Administration (DMSA) from the Ministry of Health in Vietnam. All respondents agreed to participate in the study by completing and returning an online questionnaire. The name of the participants and their organizations were anonymous.

## Results

### Number of responses

From December 2017 to January 2018, we received 621 responses from the Heads of the Pharmacy Departments in hospitals for the first questionnaire and 596 responses from clinical pharmacists for the second questionnaire. After removing duplicate responses, responses from community centers without beds, control and prevention centers, there were 560 and 570 eligible responses for the first and second questionnaire, respectively. The overall response rate of the first questionnaire was 41.2% from 1359 invited hospitals. The profile of the participating hospitals has been described in our previous publication [17].

### Demographic profile of participating hospitals

The rate of response was highest from national hospitals (57.4%) and lowest (14.8%) from private hospitals (Table 1). Most of the participated hospitals are general (71.4%), public (95.2%), and not affiliated with a university (98.9%). The majority of the responses were obtained from the North and the Mekong Delta area (a part of the Southern area of Vietnam) at 63.0 and 25.6%, respectively***.***

**Table 1:** Profile of participating hospitals

***Hospital Information***	***Number of responses*** ***N (%)***	***Total number of hospitals***^†^	***Response rate***
Hospital level (*n* = 560)			
National	27 (4.8)	47	57.4
Provincial	211 (37.7)	419	50.4
District	285 (50.9)	684	41.7
Private	27 (4.8)	182	14.8
From other Ministries/Branches	10 (1.8)	27	37.0
Hospital class (*n* = 560)			
Special class	3 (0.5)	NA^‡^	
Class 1	59 (10.5)	NA	
Class 2	179 (32.0)	NA	
Class 3	308 (55.0)	NA	
Class 4	11 (2.0)	NA	
Hospital types (*n* = 560)			
General	400 (71.4)	NA	
Specialized	160 (28.6)	NA	
Hospital funding (*n* = 560)			
Public	533 (95.2)	1177	45.3
Private	27 (4.8)	182	14.8
Area (*n* = 560)			
Red river delta	124 (22.1)	303	40.9
Northern midlands and mountainous	114 (20.4)	221	51.6
North central and south central coast	114 (20.4)	345	33.0
Central highlands	36 (6.4)	90	40.0
Southeast	29 (5.2)	191	15.2
Mekong river delta	143 (25.5)	209	68.4
Nominal beds (*n* = 560)			
≤100	204 (36.4)	–	
101–500	304 (54.30	–	
501–1000	40 (7.1)	–	
1001–1500	9 (1.6)	–	
> 1500	3 (0.5)	–	

^†^The number was extracted from the Health Statistics Year Book 2015 (published in 2017) of Ministry of Health [18]

^‡^ NA: Data were not available

### Clinical pharmacy workforce in the participating hospitals

The workforce of the participating hospitals and pharmacy departments was analyzed by hospital class (Table 2). The data indicates that the number of physicians, pharmacists and pharmacists in clinical pharmacy per 100 beds of Special Class and Class 1 hospitals were significantly lower in comparison to Class 2 and Class 3. An opposite trend was observed in the number of nurses per 100 beds. However, the numbers of full-time equivalent (FTE) clinical pharmacists per 100 beds were not significantly different among all hospital classes (*p* = 0.057, Kruskal-Wallis rank-sum test). The number of clinical pharmacists in all hospital classes was significantly lower compared to the number of physicians and nurses. The median number of pharmacists in the Clinical Pharmacy division was 1.8 and the number of FTE was 0.4, which indicates that the majority of pharmacists worked in clinical pharmacy on a part-time basis.

**Table 2:** Clinical pharmacy workforce in the participating hospitals (*n* = 560)

Characteristics (number/100 beds)	Special class* (***N*** = 3)	Class 1* (***N*** = 59)	Class 2 * (***N*** = 179)	Class 3* (***N*** = 308)	Class 4* (***N*** = 11)	Total* (***N*** = 560)	***P***-value
Pharmacists	0.8 (0.9–1.0)	1.5 (1.0–1.8)	1.9 (1.3–2.8)	2.6 (1.7–4.2)	2.0 (1.5–4.2)	1.9 (1.2–3.0)	< 0.001
Pharmacy technicians	1.9 (1.8–2.5)	2.6 (1.6–3.6)	4.0 (2.7–5.8)	5.8 (3.3–8.3)	7.5 (3.7–15.0)	4.0 (2.5–6.0)	< 0.001
Physicians	19.2 (11.1–29.8)	24.2 (19.4–29.7)	20.8 (16.4–27.4)	21.6 (16.6–29.4)	23.3 (20.0–34.3)	21.8 (16.7–28.9)	0.09
Nurses	83.1 (62.0–95.9)	55.2 (45.5–64.4)	45.3 (34.9–59.2)	36.0 (27.2–50.0)	33.3 (30.0–44.0)	41.1 (30.0–57.1)	< 0.001
Pharmacists in clinical pharmacy	0.3 (0.3–0.4)	0.6 (0.4–0.8)	1.0 (0.6–1.6)	1.3 (0.8–2.0)	0 (0–1)	1.8 (1.0–2.7)	< 0.001
FTE clinical pharmacists /100 beds	0.2 (0.1–0.2)	0.3 (0.2–0.5)	0.3 (0.2–0.7)	0.5 (0.2–0.9)	0 (0–1)	0.4 (0.2–0.8)	0.057

* Median (IQR)

### Establishment of Clinical Pharmacy Division

The majority of the participating hospitals have established Clinical Pharmacy Divisions (78.8%) (Table 3), with a small number of hospitals did not have any established clinical pharmacy activities (3.0%).

**Table 3:** Establishment of Clinical Pharmacy Division

Status	Special class (***N*** = 3)	Class 1 (***N*** = 59)	Class 2 (***N*** = 179)	Class 3 (***N*** = 308)	Class 4 (***N*** = 11)	Total (***N*** = 560)
Officially established	3 (100%)	56 (94.9%)	148 (82.7%)	229 (74.4%)	5 (45.5%)	441 (78.8%)
Not established, but still provides clinical pharmacy activities	0 (0%)	2 (3.4%)	21 (11.7%)	57 (18.5%)	2 (18.2%)	82 (14.6%)
Not established with no clinical pharmacy activity	0 (0%)	1 (1.7%)	2 (1.1%)	12 (3.9%)	2 (18.2%)	17 (3.0%)
Others	0 (0%)	0 (0%)	8 (4.5%)	10 (3.2%)	2 (18.2%)	20 (3.6%)

### Non-patient specific activities of clinical pharmacists

**Fig. 1:**
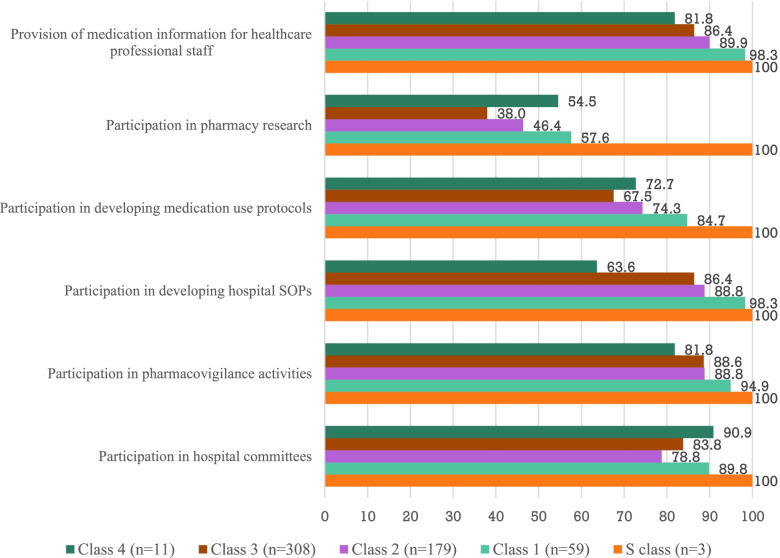
Extent of non-patient-specific activities of clinical pharmacists

Figure 1 shows the types of *non-patient-specific activities* of clinical pharmacists and highlights the differences in the extent of activities according to the hospital class. The activities of clinical pharmacists that were provided on a regular basis (“Usually” and “Always” responses) in most hospitals are participation in pharmacovigilance activities (89.3%), developing Standard Operating Procedures (SOPs) in hospitals (88.0%), providing medication information for healthcare professional staff (88.0), and participation in hospital committees (83.0%). The participation of clinical pharmacists in developing medication use protocols and pharmacy research were carried out in fewer hospitals, with 71.8 and 43.4% of responses, respectively.

### Patient-specific activities of clinical pharmacists

**Fig. 2:**
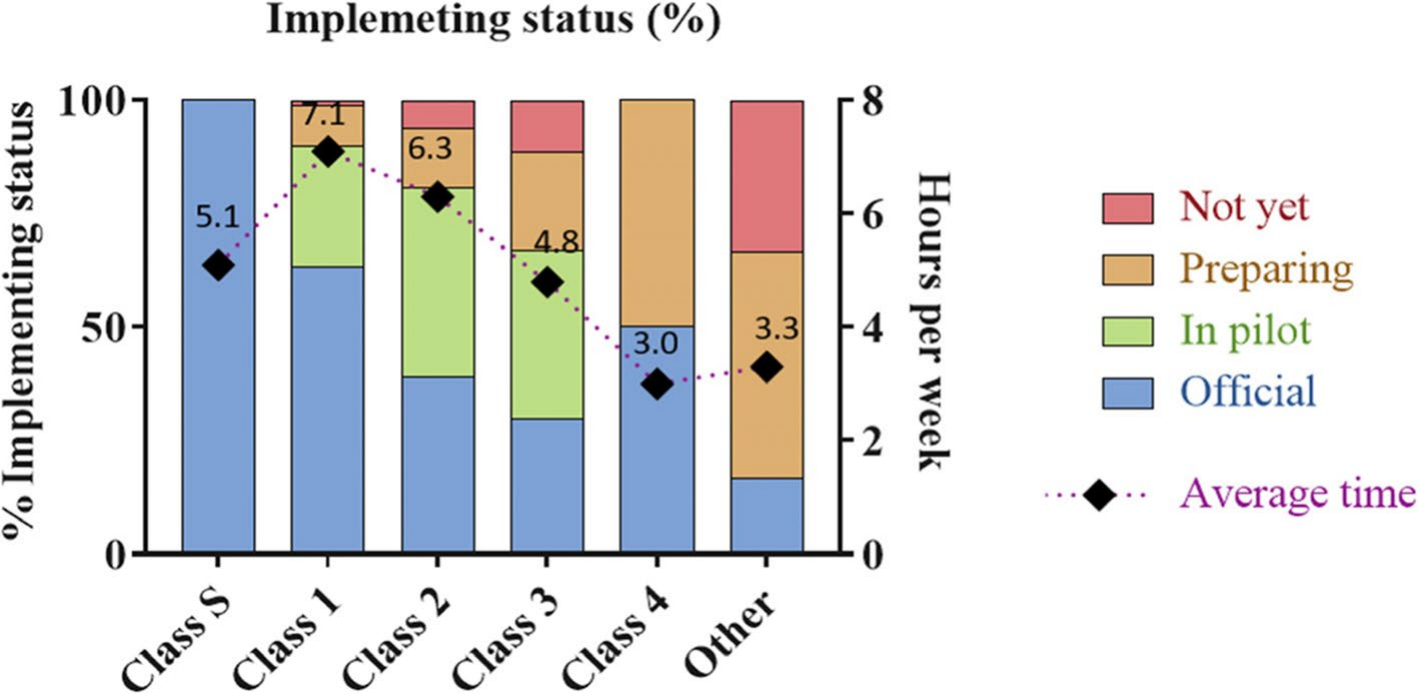
Current status of implementing patient-specific activities

According to Circular 31, *patient-specific* or *patient–centered care activities* are expected to be performed by clinical pharmacists during their ward activities. However, despite the high extent of non-patient-pecific activities, only 39.9% of clinical pharmacists reported that *patient-centered activities* were officially implemented in their hospitals (Fig. 2). The implementation rate was significantly different between Special Class hospitals (100%) and other hospital classes (less than 63.4%). More than one-third (35.9%) of hospitals were in the pilot period of implementation. In addition, the average time that clinical pharmacists spent on these activities were approximately 5.8 hours per week (Fig. 2).

**Fig. 3:**
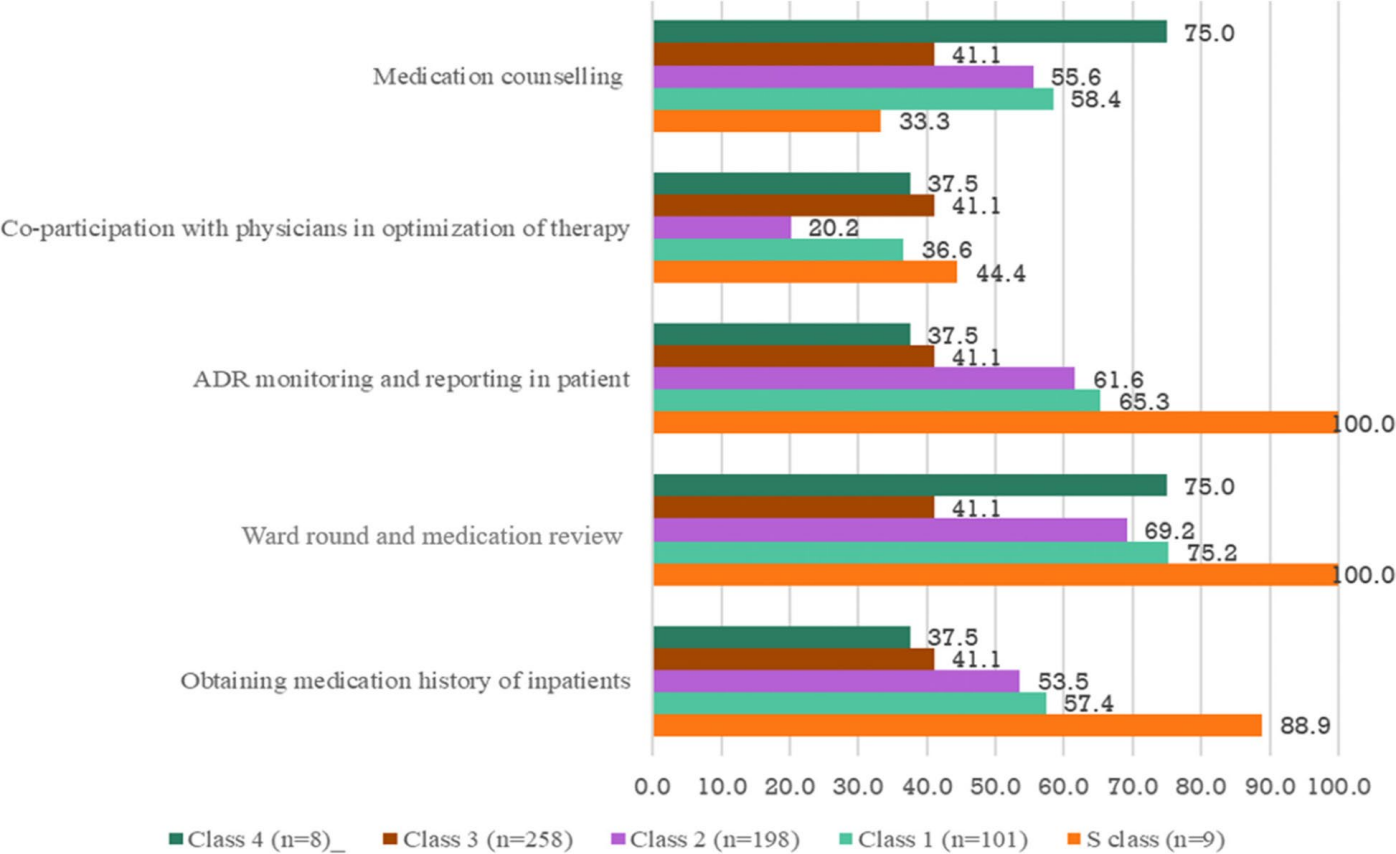
Extent of patient-specific activities of clinical pharmacists

Clinical pharmacists reported that they frequently participated in ward rounds and medication reviews for patients (64.8%) and provided medication counselling services for patients and nurses (55.7%) (Fig. 3). However, only 20.6% of clinical pharmacists collaborated with physicians to rationalize patients’ therapeutic regimens. The results also demonstrate a significant difference in the level of implementation of patient-centered activities among hospital classes, which was reflected across all aspects of the activities (Fisher’s Exact test, *p* < 0.05) (Fig. 3).

## Discussion

To the best of our knowledge, this is the first comprehensive national survey of clinical pharmacy activities in the hospital setting in Vietnam. The response rate from the hospitals was acceptable (41.2%), with the highest response from Class 2 and Class 3 hospitals. The diverse characteristics of the participating hospitals in terms of geographical location, hospital class, and type of institute (public/private) suggest that the results reflect the current pattern of clinical pharmacy activities in Vietnam. Hence, the results from this study are expected to provide helpful information for developing the National Decree of Clinical Pharmacy and the National Guideline of Implementing Clinical Pharmacy Services in Vietnamese hospitals.

The survey results highlighted a severe shortage of human resources for clinical pharmacy activities in hospital settings across all classes in Vietnam, with only 0.4 FTE clinical pharmacists per 100 beds. The number of clinical pharmacists in all hospital classes was also significantly lower compared to the number of physicians and nurses (1.8 versus 21.8 and 41.1, respectively). The constraint of limited resources has led to Vietnamese hospitals focusing their resources on the implementation of *non-patient-specific* activities. Consequently, pharmaceutical care (*patient-specific*) activities have not been well established in many hospitals. Significant differences in the availability of clinical pharmacy activities were also reported across the hospital classes, with a much higher level and extent of activities available in “higher class” hospitals.

The workforce indicators from the survey may reflect limitations in the implementation extent of the clinical pharmacy activities in Vietnamese hospitals. To provide core CPS (including medication information, ADR management, medication review, and medication reconciliation on hospital admission), a minimum number of three clinical pharmacists per hospital has been recommended by some professional associations and mandated by the law in some countries [9, 25]. Based on this recommendation, only a few Vietnamese hospitals in the Special Class category achieved the standard. The human resource issue for clinical pharmacy activities in Vietnamese hospitals has barely improved during the last several years. Previous studies have reported 0.36 FTE clinical pharmacists per 100 beds from an earlier survey in Hanoi [14] and 0.67 FTE clinical pharmacists per 100 beds in Ho Chi Minh City [15].

To account for the current low level of human resources for clinical pharmacy activities in Vietnam, several issues should be considered. There is no explicit legal requirement for the minimum number of clinical pharmacists as well as the core clinical pharmacy tasks in Vietnam [11, 12]. In addition, clinical pharmacy in hospitals is still in the initial development stages in Vietnam. The lack of research conducted in Vietnam to provide evidence of direct positive benefit, especially financial benefit from clinical pharmacy activities to the hospitals, may impede the expansion of these activities.

Congruent with the low human resources available, our survey showed that current clinical pharmacy activities in Vietnam focused primarily on process-related services in all hospital classes. *Non-patient specific services* are defined as pharmacists’ activities that are not directly related to patient care but have a significant impact on improving the quality use of medicines for patients. These activities include participating in policy development (e.g., participation in hospital committees, developing SOP and medication use protocols), research, and feedback. The participation of pharmacists in these activities demonstrates the shift in the pharmacist’s role from dispensing and supplying drugs to taking part in ensuring and improving the quality use of medicine. These particular activities can be undertaken by a small number of clinical pharmacists, which may be why *non-patient-specific activities* were considered the priority activities of clinical pharmacists in Vietnam and were highly implemented in Vietnamese hospitals irrespective of hospital class.

Furthermore, the results from the study indicated a limited level of implementation of *patient-specific activities* provided by clinical pharmacists in Vietnamese hospitals. Only ~ 40% of clinical pharmacists reported that *patient-specific activities* were officially implemented in their hospitals, despite the legal recommendation of Circular Number 31. A possible explanation is that Circular Number 31 is not considered a mandatory requirement that hospitals have to follow. Therefore, different hospitals would have different implementation plans and resources depending on their roadmap for developing clinical pharmacy services. Furthermore, our findings showed that clinical pharmacists in Vietnam only spend an average of 5.8 hours per week performing these duties, which is significantly different from the clinical pharmacy models in developed countries. With such a limited amount of time dedicated to *patient-specific activities*, medication counselling (57%), ADR monitoring and reporting (57.5%), and obtaining medication history of inpatients (49%) were reported to be the most commonly performed activities by the clinical pharmacists. Meanwhile, the core activity of pharmaceutical care (co-participation with physicians in optimization of therapy) was performed regularly by only one-fifth of the participating clinical pharmacists. It should be noted that the “usually” and “always” responses in the survey regarding the core activities need to be interpreted relative to the low average time (5.8 hours per week) that the clinical pharmacists had on the ward.

Barriers to the implementation of pharmaceutical care services in some countries, including developing countries similar to Vietnam, have been reported in studies from Brazil [26, 27], Nigeria [28], Lebanon [29], Kuwait [30], Portugal [31], and China [32]. A systematic review conducted by Onozato et al. [33] also identified the multifactorial nature surrounding the implementation process of clinical pharmacy services in hospitals, with the most cited influencing factors related to the pharmacists, healthcare team, local hospital, and national organization. More specifically, the major barriers related to the pharmacists were their mindset, hard to shifting the role, lack of readiness, and inadequate clinical education/training [26, 31]. Barriers at the organizational level include insufficient human resources, difficulty in collaboration between pharmacists and other healthcare staff, lack of support by hospital leaders, and lack of awareness by other healthcare staff [30, 31]. Our present study suggests that these identified barriers may also apply in the Vietnamese context, including limited human resources (discussed above), inadequate clinical training and the lack of an official Standard of Practice for clinical pharmacy activities. Along with a lack of human resources, another significant issue in Vietnam is a dearth of clinical pharmacist training. In a 2011 survey of 137 clinical pharmacists, nearly 40% indicated that they were not trained in clinical aspects of pharmacy in college and only 58% reported participating in continuing education courses [34]. Additionally, the lack of an official Standard of Practice for the provision of *patient-specific activities* in the whole country may be one of the main reasons these activities have not been implemented systematically. However, further studies are needed with larger numbers of interviewees to comprehensively understand the barriers to pharmaceutical care activities in the Vietnamese hospital setting.

Regarding the strengths and limitations of our study, this is the first national survey focused on clinical pharmacy practice in the hospital setting in which all hospitals in Vietnam were invited to participate. Circular 31 and Pharmaceutical Law 2016 related to clinical pharmacy activities were employed to design the survey questionnaires, thus allowing the elicitation of the impact of these legal requirements on current clinical pharmacy activities in Vietnam. Nevertheless, the survey results should be considered in the context of the study limitations. Firstly, it was a self-administered survey where the respondents could have potentially misunderstood the questions but did not have the opportunity to clarify with the researcher. The study used the Likert scale with relative frequency, which also could lead to different understanding by the respondents. Furthermore, there may be some self-selection bias leading to overestimation as clinical pharmacists who are more confident in practicing pharmaceutical care may be more willing to participate. Finally, the explanation for some of the barriers affecting the extent of clinical pharmacy activities was hypothesized by the research team. Therefore, further studies focusing on the difficulties and advantages of the implementation of clinical pharmacy activities in Vietnam are required to confirm our suggested explanations.

## Conclusion

The study provided an overview of the current status of clinical pharmacy activities in Vietnamese hospitals. These activities were implemented at a much lower level in Vietnam than developed countries. In general, the extent of implementation of clinical pharmacy activities varied based on the type of activity and classification of the hospital in Vietnam. The extent of these activities was more established in higher class hospitals with a larger number of clinical pharmacists. In addition, the current implementation status focused more on *non-patient specific* activities, while *patient-specific* activities remained insufficiently established in Vietnam. Therefore, further research focusing on the enablers and barriers to the implementation of clinical pharmacy services from the perspective of stakeholders is required to provide a more comprehensive understanding and solutions for better practice.

## Supplementary Information

**Additional file 1.** Part 1. Workforce and Non-patients Specific Activities (for the Head of Department of Pharmacy).

**Additional file 2.** Questionnaire of the national survey Part 2 – Patient – specific activities (for the clinical pharmacists).

## Data Availability

All data generated and analyzed during the current study are available from the corresponding author on reasonable request.

## Abbreviations

ADRs: Adverse Drug Reactions
MOH: Ministry of Health
DMSA: Department of Medical Services Administration
FTE: Full-time Equivalent
