# Supplementary material for: Implementing clinical pharmacy activities in hospital setting in Vietnam: current status from a national survey
Source: BMC Health Serv Res. 2022 Jul 7;22:878. doi: 10.1186/s12913-022-08242-5 (PMC9264624; doi:10.1186/s12913-022-08242-5)
Supplement: Supplementary file 1 — Additional file 1. Part 1. Workforce and Non-patients Specific Activities (for the Head of Department of Pharmacy). [file 12913_2022_8242_MOESM1_ESM.docx]

**Supplementary file 1**

**Part 1. Workforce and Non-patients Specific Activities**

**(for the Head of Department of Pharmacy)**

**Section 1. General information and workforce**

1.1. Name of hospital

1.2. Hospital location (province)

1.3. Level of hospital

1. National hospital
2. Regional hospital
3. Provincial hospital
4. Other: ………………………….

1.4. Class of hospital

1. Special class
2. Class 1
3. Class 2
4. Class 3
5. Other:…………………..

1.5. Type of hospital:

1. General hospital
2. Specialize hospital
3. Other:…………………..

1.6. Sources of funding

1. Private
2. Public
3. Other:…………………

1.7. Affiliations

1. Belong to a university
2. Not belong to a university

1.8. Number of nominated beds in last year: ……………..

1.9. Number of pharmacists in hospital:…………

1.10. Number of doctors in hospital: ………….

1.11. Number of nurses in hopital: ………..

1.12. Number of pharmacy technicians in hospital: ……….

**Section 2. The establishment of Clinical Pharmacy (CP) Division**

2.1. The status of the establishment of CP division in hospital:

1. Official established
2. Not established, but still provides clinical pharmacy activities
3. Not established with no clinical pharmacy activity
4. Other

2.2. Number of pharmacist in the CP division?.…………….

2.3. Number of doctor in the CP division?.................

2.4. Number of FTE pharmacist in the CP division?…………….

2.5. Number of pharmacist meeting requirement criteria accroding to Circular No.31 in the CP division?…………….

**Section 3. The extent of non patient specific activities**

3.1. What is the extent of the clinical pharmacy unit/team’s participation in the following

committees’ activities in your hospital? Please choose the relevant ones.

(with 1 = never/don’t have; 2 = rarely; 3 = sometimes; 4 = usually; 5 = always; NA: do not know)

| **Committee** | **The extent of participant** | | | | | |
| --- | --- | --- | --- | --- | --- | --- |
| The Pharmacy and Therapeutic Committee | **1** | **2** | **3** | **4** | **5** | **NA** |
| The Science and Technology Committee | **1** | **2** | **3** | **4** | **5** | **NA** |
| Infection Control Group | **1** | **2** | **3** | **4** | **5** | **NA** |
| Patient Safety Group | **1** | **2** | **3** | **4** | **5** | **NA** |
| Quality Assurance Group | **1** | **2** | **3** | **4** | **5** | **NA** |
| Quality Assurance Group | **1** | **2** | **3** | **4** | **5** | **NA** |
| Nutrition Group | **1** | **2** | **3** | **4** | **5** | **NA** |
| Hygiene Management Group | **1** | **2** | **3** | **4** | **5** | **NA** |
| Other:……………………… | **1** | **2** | **3** | **4** | **5** | **NA** |

3.2. What is the extent of clinical pharmacists’ involvement in building or revising the hospital formulary? Please choose all relevant options.

(with 1 = never/don’t have; 2 = rarely; 3 = sometimes; 4 = usually; 5 = always, NA: do not know)

| **Activities** | **The exent of activities** | | | | | |
| --- | --- | --- | --- | --- | --- | --- |
| Provide evidence to compare efficacy and safety of drugs in/with hospital formulary | **1** | **2** | **3** | **4** | **5** | **NA** |
| Provide evidence to compare the cost benefit of drugs in/with hospital formular | **1** | **2** | **3** | **4** | **5** | **NA** |
| Evaluate, control the duplication of generic drugs | **1** | **2** | **3** | **4** | **5** | **NA** |
| Other: | **1** | **2** | **3** | **4** | **5** | **NA** |

3.3. What is the extent of clinical pharmacists’ participation in building the following medication use protocols and therapeutic guidelines? Please choose all relevant options.

(with 1 = never/don’t have; 2 = rarely; 3 = sometimes; 4 = usually; 5 = always, NA: do not know)

| **Activities** | **The exent of activities** | | | | | |
| --- | --- | --- | --- | --- | --- | --- |
| Building the process of medication information in hospital | **1** | **2** | **3** | **4** | **5** | **NA** |
| Building ADR monitoring protocol | **1** | **2** | **3** | **4** | **5** | **NA** |
| Building Medication Error monitoring protocol | **1** | **2** | **3** | **4** | **5** | **NA** |
| Building hospital therapeutic guidelines | **1** | **2** | **3** | **4** | **5** | **NA** |
| Building hospital technical processes | **1** | **2** | **3** | **4** | **5** | **NA** |
| Others……….. | **1** | **2** | **3** | **4** | **5** | **NA** |

3.4. What is the extent of clinical pharmacists’ participation in building medication monitoring protocols for following drug groups?

(1 = never/don’t have; 2 = rarely; 3 = sometimes; 4 = usually; 5 = always, NA: do not know)

| **Group of medicine** | **The exent of participant** | | | | | |
| --- | --- | --- | --- | --- | --- | --- |
| Narrow therapeutic window medicines | **1** | **2** | **3** | **4** | **5** | **NA** |
| Medicine with serious ADRs | **1** | **2** | **3** | **4** | **5** | **NA** |
| Antibiotics | **1** | **2** | **3** | **4** | **5** | **NA** |
| Prophylatic Antibiotics | **1** | **2** | **3** | **4** | **5** | **NA** |
| Medicines with special precaution for infusion in pediatric | **1** | **2** | **3** | **4** | **5** | **NA** |
| Medicines with special precaution for infusion in oncology | **1** | **2** | **3** | **4** | **5** | **NA** |
| Medicines with Special precaution for storage condition | **1** | **2** | **3** | **4** | **5** | **NA** |
| Medicines with Therapeutic drug monitoring (TDM) | **1** | **2** | **3** | **4** | **5** | **NA** |
| Other | **1** | **2** | **3** | **4** | **5** | **NA** |

3.5. What is the extent of clinical pharmacists’ participation in the following research activities in the hospital?

(with 1 = never/don’t have; 2 = rarely; 3 = sometimes; 4 = usually; 5 = always, NA: do not know)

| **Research activities** | **The exent of activities** | | | | | |
| --- | --- | --- | --- | --- | --- | --- |
| Drug Usage evaluation research | **1** | **2** | **3** | **4** | **5** | **NA** |
| Quality Improvement research in Pharmacy Practice | **1** | **2** | **3** | **4** | **5** | **NA** |
| Clinical Trials | **1** | **2** | **3** | **4** | **5** | **NA** |
| Protocol-Adherence Research | **1** | **2** | **3** | **4** | **5** | **NA** |
| Other | **1** | **2** | **3** | **4** | **5** | **NA** |

3.6. What is the extent of clinical pharmacists’ provision of medication for healthcare professional staff in the hospital?

(with 1 = never/don’t have; 2 = rarely; 3 = sometimes; 4 = usually; 5 = always, NA: do not know)

| **Activities** | **The exent of activities** | | | | | |
| --- | --- | --- | --- | --- | --- | --- |
| Providing MI for HCPs via paper  documents to clinical wards | **1** | **2** | **3** | **4** | **5** | **NA** |
| Updating MI for HCPs via grand  rounds in hospital | **1** | **2** | **3** | **4** | **5** | **NA** |
| Providing MI for HCPs via  pharmacy bulletins/posters in the  hospital | **1** | **2** | **3** | **4** | **5** | **NA** |
| Updating MI for clinical wards via  LAN | **1** | **2** | **3** | **4** | **5** | **NA** |
| Updating MI for HCPs via  scientific conferences/seminars in  hospitals* | **1** | **2** | **3** | **4** | **5** | **NA** |

3.7. What is the extent of clinical pharmacists’ participant in the pharmacovigilance activities?

(with 1 = never/don’t have; 2 = rarely; 3 = sometimes; 4 = usually; 5 = always, NA: do not know)

| **Activities** | **The exent of activities** | | | | | |
| --- | --- | --- | --- | --- | --- | --- |
| Being coordinator in reporting  ADRs of hospital | **1** | **2** | **3** | **4** | **5** | **NA** |
| Participating in monitoring and  supervise ADRs in hospitals | **1** | **2** | **3** | **4** | **5** | **NA** |
