# Supplementary material for: Implementing clinical pharmacy activities in hospital setting in Vietnam: current status from a national survey
Source: BMC Health Serv Res. 2022 Jul 7;22:878. doi: 10.1186/s12913-022-08242-5 (PMC9264624; doi:10.1186/s12913-022-08242-5)
Supplement: Supplementary file 2 — Additional file 2. Questionnaire of the national survey Part 2 – Patient – specific activities (for the clinical pharmacists). [file 12913_2022_8242_MOESM2_ESM.docx]

**Supplementary file 2.**

**Questionnaire of the national survey**

**Part 2 – Patient – specific activities**

**(for the clinical pharmacists)**

**Section 1. General information of hospital**

1. Name of hospital

2. Hospital location (province)

3. Level of hospital

- 1. National hospital
  2. Regional hospital
  3. Provincial hospital
  4. Other………………………….

4. Class of hospital

1. Special class
2. Class 1
3. Class 2
4. Class 3
5. Other:…………………..

5. Type of hospital

- 1. General hospital
  2. Specialize hospital
  3. Other:…………………..

6. Sources of funding

- 1. Private
  2. Public
  3. Other:…………………

7. Affiliations

1. Belong to a university
2. Not belong to a university

**Section 2. The extent of patient-specific activities**

2.1. In your hospital, what is the status of clinical pharmacy services in clinical wards?

1. Have been officially implemented
2. In the pilot period of implementation
3. Have not been implemented yet, but we have prepared for these actitivites
4. Other:………………….

2.2. What is the extent of your clinical activities in each of clinical ward below? Please choose all relevant options.

(1 = never/don’t have; 2 = rarely; 3 = sometimes; 4 = usually; 5 = always)

| **Ward** | **1** | **2** | **3** | **4** | **5** | **Others** |
| --- | --- | --- | --- | --- | --- | --- |
| Critical Care |  |  |  |  |  |  |
| Anti-toxic |  |  |  |  |  |  |
| Infections |  |  |  |  |  |  |
| Gynecology/Nephrology |  |  |  |  |  |  |
| Gatroenterology |  |  |  |  |  |  |
| Cardiology |  |  |  |  |  |  |
| Oncology |  |  |  |  |  |  |
| Radiology |  |  |  |  |  |  |
| Geriatric |  |  |  |  |  |  |
| Pediatric |  |  |  |  |  |  |
| Endocrinology |  |  |  |  |  |  |

2.3. Please estimate the average (and the min-max) time of your clinical activities per weeks. For examples, 12 hours/week (min 2h – max 20h)

…………..

2.4. What is the availability of criteria of patient in the clinical ward that you choose to take care?

1. Using the official criteria for target patients
2. Available criteria for target patients but not using
3. Not available

If the answer is A, could you please list some of criteria?

………………………………………………

If the answer is B, why do you not use them?

………………………………………………..

2.5. In clinical wards, which following activities that you provide for patients and healthcare staffs?

(you can choose many options) :

(1 = never/don’t have; 2 = rarely; 3 = sometimes; 4 = usually; 5 = always)

| **Activities** | **1** | **2** | **3** | **4** | **5** | **Other** |
| --- | --- | --- | --- | --- | --- | --- |
| Obtaining medication history of inpatients/medication reconciliation |  |  |  |  |  |  |
| Medication review |  |  |  |  |  |  |
| ADR monitoring and reporting in patient |  |  |  |  |  |  |
| Co-participation with physicians in optimization of therapy |  |  |  |  |  |  |
| Medication counselling |  |  |  |  |  |  |

2.6. Related to Medication reconciliation activities (obtaining medication history), how do you do following activities (you can choose many options) :

(1 = never/don’t have; 2 = rarely; 3 = sometimes; 4 = usually; 5 = always)

| **Activities** | **1** | **2** | **3** | **4** | **5** | **Other** |
| --- | --- | --- | --- | --- | --- | --- |
| *Information that you obtain from patients* | | | | | | |
| Medication history |  |  |  |  |  |  |
| Medical history |  |  |  |  |  |  |
| Allergic history |  |  |  |  |  |  |
| Medication adherence |  |  |  |  |  |  |
| *The sources that you use to obtain medication history* | | | | | | |
| Paper-based medical records |  |  |  |  |  |  |
| Electronic-based medical records |  |  |  |  |  |  |
| Interviewing patients and relatives |  |  |  |  |  |  |

2.7. Related to medication review and ward round, which following activities that you have done (you can choose many options) for patients:

(1 = never/don’t have; 2 = rarely; 3 = sometimes; 4 = usually; 5 = always)

| **Activities** | **1** | **2** | **3** | **4** | **5** | **Others** |
| --- | --- | --- | --- | --- | --- | --- |
| Check indications |  |  |  |  |  |  |
| Check contra-indications |  |  |  |  |  |  |
| Evaluate medicines choice |  |  |  |  |  |  |
| Evaluate dosage for each medicine |  |  |  |  |  |  |
| Evaluate administration route for each medicine |  |  |  |  |  |  |
| Evaluate administration time for each medicine |  |  |  |  |  |  |
| Evaluate the interval time for each medicine |  |  |  |  |  |  |
| Note the information relevant to adverse drug reactions, drug allergy |  |  |  |  |  |  |
| Check drug interactions |  |  |  |  |  |  |
| At this time, none of above has been done |  |  |  |  |  |  |
| Other: |  |  |  |  |  |  |

2.8. During the treatment, which following activities that clinical pharmacists have done in

collaboration with physicians to optimize patients’ therapy (you can choose many options):

(1 = never/don’t have; 2 = rarely; 3 = sometimes; 4 = usually; 5 = always)

| **Activities** | **1** | **2** | **3** | **4** | **5** | **Other** |
| --- | --- | --- | --- | --- | --- | --- |
| Identify possible drug-related problems |  |  |  |  |  |  |
| Set the therapy goals and propose solutions for problems with physicians |  |  |  |  |  |  |
| Follow patients’status based on clinical symtoms |  |  |  |  |  |  |
| Monitor changes in laboratory tests’ results |  |  |  |  |  |  |
| Suggest doing additional laboratory tests if needed |  |  |  |  |  |  |
| Monitor ADRs and drugs’ toxicity |  |  |  |  |  |  |
| Suggest the interventions with physicians |  |  |  |  |  |  |
| At this time, none of above has been done |  |  |  |  |  |  |
| Other: |  |  |  |  |  |  |

2.9. Related to medication conselling, how often do you provide each of following activitied?

| **Actitivities** | **1** | **2** | **3** | **4** | **5** | **Other** |
| --- | --- | --- | --- | --- | --- | --- |
| Advice for nurses on how to admisnister medicines |  |  |  |  |  |  |
| Advice for inpatients on how to take medicine properly during hospital stay |  |  |  |  |  |  |
| Advice for inpatients on how to take medicine properly at discharge |  |  |  |  |  |  |
| Advice for outpatients on how to take medicine properly |  |  |  |  |  |  |
| Other:  ………………………………………… |  |  |  |  |  |  |

14. Which following medication groups and how often do clinical pharmacists counsel for

healthcare professionals in clinical wards:

(1 = never/don’t have; 2 = rarely; 3 = sometimes; 4 = usually; 5 = always)

| **Committee** | **1** | **2** | **3** | **4** | **5** | **Other** |
| --- | --- | --- | --- | --- | --- | --- |
| Antibiotics |  |  |  |  |  |  |
| Central nervous system agents |  |  |  |  |  |  |
| Anticoagulant agents |  |  |  |  |  |  |
| Immunodepressants (cyscloporin,  tacrolimus..) |  |  |  |  |  |  |
| Total parenteral nutrition (oral or enteral  route) |  |  |  |  |  |  |
| Antineoplastic |  |  |  |  |  |  |
| Anti-diabetes medicines |  |  |  |  |  |  |
| Cardiovascular medicines |  |  |  |  |  |  |
| Other |  |  |  |  |  |  |

2.10. Which following methods do you document the clinical pharmacy intervention (You can choose many options):

1. Not documented
2. Paper-based documentation using official forms
3. Paper-based documentation without official forms
4. Computer-based documentation, using software i.e Excel
5. Internet-based documentation (i.e Google Form)
6. Specialized software for documentation
7. Others………………………
